# Child Welfare and Nutrition

**Published:** 1920-12-25

**Authors:** 


### Child Welfare and Nutrition.

### THE PREVALENCE OF RICKETS.

Some recent contributions to the literature of vitamines are analysed in the latest issue of the *International Journal of Public Health*; and the discussion carried on with regard to the fat-soluble vitamine brings out with striking emphasis the relation between child-welfare and nutrition. There seems no doubt, as is here pointed out, that the disease of rickets has become more prevalent, and that the nutritional factor enters largely into this.

Rickets is a disease characterised in its later stages by very marked bone changes, of which the most prominent and constant are enlarged epiphyses, followed by softening of bones and subsequent bending. It is rare in children that are breast fed, "so rare as to seem to justify the conclusion that where this does occur the mother's food was so lacking in this vitamine that her milk was also devoid of it." It is common in children fed on cow's milk, especially—and this is a point to which the attention of mothers should be particularly directed—on cow's milk that has been diluted with water, and "so-called" humanised milk. The

**THE PUBLIC WELL-BEING**—(*continued*).

withholding also of butter from the diet of young children and the substitution of margarine may be an important factor in rickets. It is common in children fed on farinaceous food, so common as to give rise to an impression still very prevalent that "rickets is due to a shortage of fats *per se*." The condition is much predisposed to by the want of light and air in many of our large towns. It has become much more prevalent, especially in certain towns—for instance, Vienna—since the war. Here the conditions as to light and air have remained constant, but the difficulty of obtaining fats rich in fat-soluble A vitamine has greatly increased. "The condition of rickets, which is commonly initiated in children under two years of age, tends to a spontaneous cure with no treatment, though the deformities persist." It seems to be a disease of early youth, due to a shortage of a material that is acutely needed up to two years. After that time it is needed in so small a quantity that children can thrive for a long time on food containing only a trace of it. Its cure is much speeded by the administration of food rich in the fat-soluble vitamines (butter, cod-liver oil, etc.). In the older literature there was a condition described as acute rickets which is now identified with infantile scurvy.

The conclusions of an extensive consideration of the subject are that recent contributions to literature seem to point definitely to a nutritional origin for rickets and late rickets. The shortage is due to the lack, not of quantity, but of quality of the fat provided. Certain fats, particularly butter fat and cod-liver oil, provide this factor in large amounts. The immense increase of rickets in Europe during the war is out of all proportion to the deterioration of housing and other environmental factors. The depreciation of available food is unquestionable, and the shortage is just in those fats that contain the greatest proportion of the fat-soluble vitamine.

